# On the Treatment of Spermatorrhœa by Cauterization of the Urethra, with Cases

**Published:** 1850-05

**Authors:** 


### *On the Treatment of Spermatorrhœa, by Cauterization of the Urethra, with Cases.* By F. P. Porcher, M. D.

Case 1st.—T. B., a gentleman, æt. 19, who had been unsuccessfully treated for what had been vaguely termed dyspepsia, applied to us in July, 1849. He appeared to suffer under a severe form of disease, attended with very distressing concomitants, viz : feeble pulse, emaciation, sleepless nights, cold extremities, occasional diarrhœa, and extreme low spirits, which took on the form of a religious melancoly. The patient was very much prostrated, and so reduced in strength as to give just cause to apprehend an immediate fatal termination, as there was also some evidence of softening of the medullary substance of the brain and spinal column, indicated, among other symptoms, by dilated pupils, impairment of the mental powers and inability to fix the attention on any one subject. Hysterical attacks became frequent, and occasionally during the night there was present some evidence of a condition resembling catalepsy. We have, repeatedly, in the presence of others, seen him, after retiring to bed, seem apparently asleep, from which he could with difficulty be aroused, and remaining with the eye-lids open, without closing them at all, for twenty minutes or more ; the balls staring fixedly, but vacantly to the ceiling. There was an inconquerable desire to remain alone, and be left to the enjoyment of complete solitude, from which no persuasion could draw him off. His disposition was wayward and uncertain, and he was constantly settling scruples of conscience on every occasion, however trivial and unimportant.

At a much earlier date, a cup of moderately strong tea, or coffee, was sufficient to unnerve him, or lead his thoughts into the most fantastic channels. On these occasions, he would become the " hero of self-imaginings ;" his excited fancy conjuring up the most remarkable transactions, in which he was chief actor.

A physician of eminence in this city, with whom we saw him in consultation, differing from us in viewing it as an affection dependent upon derangement of the portal circulation, prescriped moderately low diet, with the use of arrow root and port wine, citrate of iron and quinine powders, an occasional blue pill at night, and counter-irritation to the abdomen with croton oil liniment, This course being productive of no benefit, and the patient all the while rapidly sinking, we determined upon our own responsibility, to give him the benefit of Prof. Lallemand's operation.

Our reasons for taking a different view of the case, were the following : From suggestions in the work of the author alluded to, upon inquiring whether he had ever suffered from ascarides, we learned that three years previously, whilst at school, he had for some time noticed in his evacuations a large number of the small worms which sometimes infest the rectum, but which at the time attracted only a passing attention. The presence of these was, however, accompanied with such determination toward the genital organ, as to induce frequent and weakening nocturnal emissions.—These continued, and after a while gradually disappeared as the individual grew weaker and more liable to the effects of irritation and nervous excitability. During the whole of the interval between the periads alluded to, embracing some three years, the patient was engaged at his studies, closely attending to his duties, and daily becoming worse, until from sheer inability to proceed, he was forced to discontinue application to books, and to place himself uuder our care, when we found him in the condition described above.

From careful examinations and minute inquiry, we were convinced of the connection of cause and effect ; and, attributing all the distressing sequelæ to the constant and wasting drain on the system silently going on, combined with the general impairment of the nervous functions from continued mental activity, contributing to induce dyspepsia, palpitation of the heart, irreguiarity of the bowels, anemia and the most distressing melanancholia, we determined to restore the tone, or relieve irritation, of the prostatic portion of the urethra. By this means arresting the diurnal emissions, which, though we failed with the microscope in detecting spermatozoa in the urine, yet we conceived to be present ; they, according to the views of M. Lallemand, having succeeded upon the disappearance of the nocturnal. The diagnosis was completely and satisfactorily confirmed by the entire success of the operation ; notwithstanding the advanced stage of the disease, gave us little expectation of accomplishing a great deal.

In the presence of a medical friend, who had been himself a pupil of M. Lallemand's, and who fully testifies to the success of his practice, we proceeded to ascertain the length of the urethral canal, by introducing a graduated gum elastic catheter ; upon the withdrawal of which, after having first directed the patient to empty his bladder, the cauterizing instrument was introduced, its mouth passed into the bladder, and the loaded point unsheathed, which being drawn rapidly outwards for two inches before re-sheathing it, left the orifices of the seminal ducts and the walls of the lower fifth of the urethra in contact with the caustic. A few drops of blood accompanied the withdrawal of the instrument ; the patient complained of pain, an immediate desire to go to stool and to void urine, which was tinged with blood ; the latter escaping *guttatim* for several hours.—The soreness lasted for some days, and the patient began rapidly to improve. The recurrence of normal and sensible nocturnal emissions, with discharge of spermatozoa, and the disappearance of the chain of morbid symptoms, having their origin in the testicles and the organs related to it, were some of the earliest evidences of improvement.

In the course of three weeks, some signs of relapse having manifested themselves, we repeated the operation, when they immediately began to disappear. Many months having now elapsed, he is apparently perfectly restored ; weighs more than he has ever done ; and is, in reality, in more robust health than at almost any period of his life ; with a tendency to corpulency. He has regained his vivacity and cheerfulness of disposition, and has returned to his usual pursuits, in all of which he takes an interest; enjoying life as much as most men. His dyspeptic symptoms, we must also add, have for the most part disappeared.

This account is drawn in no high colors ; the rapidity of the recovery, which actually seemed marvellous, and which our associate unites with us in ascribing entirely to the operations, so immediately consequent upon them was it, and besides which, nothing else could have saved him, will not allow us to abate any thing from the entire confidence conveyed by the above description.

Case 2d.—F. S., a gentleman aged 21, applied to us in 1840, complaining of general bad feeling, low spirits and occasional attacks of melancholy, so distressing as at times he confessed to induce him to wish to put an end to his life ; he took pleasure in few of his former enjoyments, and found his memory and intellect so much impaired as to be unable to confine attention for any length of time to books. He also gradually became thin and wasted, almost lost his virility, and apprehended perfect impotence.—There were, in addition, the usual symptoms ascribed to dyspepsia, and at times, a tendency to constipation. His appetite was moderate, but he had to be careful of his diet, abstaining from tea, coffee and wine ; the genital organs were flaccid and relaxed, with some enlargement of the epididymis and cord ; the urethral orifice was abnormally red, and exhibitied signs of irritation extending along its course to the bladder. The patient himself attributed all his ailments to excessive masturbation, in which he had indulged for some years, not seeming to have been aware of its injurious effects. We prescribed the red precipitate of iron, in powder, with small doses of the tinct. of bark and an occasional blue pill ; moderately cold applications were made to the genitals and spinal column, and the avoidance of all sources of excitement advised. These measures, however, failed in restoring him to health, though persisted in for several months.—The gentleman had tried ineffectually sarsaparilla syrups, the Juno cordial, and other secret preparations.

At intervals of two months we cauterized the lower third of the urethra, as in the first case, of which, however, this had priority in time. Since which the patient's health has improved in every respect, embracing a corresponding amelioration of all the bad symptoms related above ; he has in a great degree recovered his cheerfulness and ability to apply himself to intellectual labor ; all of which he ascribes to the action of the remedy, and without which, he asserts, life was so miserable, he must have put a violent end to it.

The failure of the first two operations to give him the immediate and marked relief characterizing those of the first case, is accounted for by the fact, that, without intending it, we had allowed the loaded extremity of the *porte caustique* to act on the side of the urethral canal, instead of twisting the curled platinum shaft, so as to make it take effect on the lower wall of the urethra, where the orifices of the seminal ducts are more directly situated. The relief was very considerable, however ; so much so, that at his urgent request, we repeated the operations. He also allowed himself to indulge in some dissipation for several months, and to this he ascribes his failure to recruit as rapidly as he might have done ; adding his own testimony to the effect, that without the general improvement of health consequent upon the remedial measures to which he subjected himself, he could never have undergone what he did subsequently. We must also be allowed to state, that a full effect was not obtained, by reason of our desire to err on the safe side, and consequently withdrawing the instrument before it acted effectually on the part. There was, therefore, not by any means so much pain and local irritation produced in this case as in the other.

*Remarks.*—With reference to the best method of introducing the instrument—a practical suggestion was brought to our notice by a London Journal, and confirmed by the difficulty which we experienced, and others may notice in cases like those under consideration ; viz : the necessity of depressing the porte caustique before it can be made to pass the prostate, which is enlarged so frequently, and to such a degree, as almost to persuade the unwary that a stricture exists. In our operations, a sudden and forcible depression of the instrument was always necessary to its successful introduction.

Although the views of M. Lallemand, formerly the distinguished Professor of Clinical Surgery at Montpelier, have met with some opposition, we must rank ourselves among those who look upon him as one who has originated and illustrated by a long course of successful practice, a method of relieving and curing an important class of disease, heretofore considered either beyond the reach of medical aid, or entirely misunderstood. Besides the testimony afforded by the cases related above, were evidence wanting, we might add that the highly respectable physician whom we before alluded to, informs us that he has seen and frequently conversed with numbers of those under the care of M. Lallemand, who from the very lowest stage of the disease were restored to health. Many of these individuals, the author himself states in his work, had been sent to him on account of suspected cerebral affection, many were supposed to suffer from chronic gastritis, from aneurisms near the heart, from nervous affections, and especially from hypochondriasis ; every symptom of which, when we are furnished with the clue, can be traced back to the disorder arising directly in, or communicated to, the generative organs. From these, a constant drain is going on in the shape of nocturnal or diurnal emissions, and the whole train of symptoms affecting the various functions ensue. Subsequently, organic alterations, compromising life, follow. The usual cause of failure in the management of these cases is owing to the practitioner treating the sequelae instead of attacking the evil at its root.

M. Lallemand divides the causes of the disease into two varieties; the one, a state of irritation remaining after venereal excesses or masturbation which induces an increased secretion and hurried discharge of the seminal fluid "without complete erection and almost without sensation," the other a relaxation of the ejaculatory canals and their orifices accompanying his, state of irritation, which allows of the escape of the semen without either erection or enjoyment ; this takes place diurnally, especially during defecation and the expulsion of the urine. The transition between these different stages of seminal evacuation is sometimes so insensiblo that it is impossible for the patient or even for the medical attendant to specify the exact period.

Case *first*, we consider of this description. The ejaculatory ducts and urethral canal become so flaccid and relaxed that the patient experiences no sensations during the passage of the spermatic fluid. The application of the nitrate of silver producing some modification in the tissues, viz: inflammation and slight cicatrization, increases the tonicity, lessens the calibre of the passages, and restores them to their original condition. Healthy secretions are the result, and the mutual relations and play of affinities of the entire system are again resumed under their ordinary and normal conditions.

M. Lallemand has been practising cauterization for twenty years, and has never seen injurious effects result from it, provided it be done rapidly; "one operation," he states, "usually sufficing." *Two-thirds of the cases of Spermatorrhœa would, in his opinion, be beyond the reach of medical assistance, were it not for the beneficial effects of nitrate of silver, applied to the prostatic portion of the urethra.* We are confident that we see daily around us, individuals who are left to die for want of its timely application. It is true, some other affections, such as diarrrhœa, epilepsy, or diseases involving the circulatory system, ultimately supervene, and to which the fatal result is ascribed; but the *punctum saliens*, or point of origin, is generally found in the generative organs, or parts from whence revulsions to them are frequent.—*Charleston Med. and Surg. Jour.*

